# Extracellular Vesicles as Biomarkers for Parkinson’s Disease: How Far from Clinical Translation?

**DOI:** 10.3390/ijms25021136

**Published:** 2024-01-17

**Authors:** Alice Gualerzi, Silvia Picciolini, Marzia Bedoni, Franca Rosa Guerini, Mario Clerici, Cristina Agliardi

**Affiliations:** 1 IRCCS Fondazione Don Gnocchi Onlus, 20148 Milan, Italy; agualerzi@dongnocchi.it (A.G.); spicciolini@dongnocchi.it (S.P.); mclerici@dongnocchi.it (M.C.); cagliardi@dongnocchi.it (C.A.); 2 Department of Pathophysiology and Transplantation, University of Milan, 20122 Milan, Italy

**Keywords:** Parkinson’s disease (PD), extracellular vesicles, exosomes, biomarkers, α-synuclein

## Abstract

Parkinson’s disease (PD) is a neurodegenerative disorder affecting about 10 million people worldwide with a prevalence of about 2% in the over-80 population. The disease brings in also a huge annual economic burden, recently estimated by the Michael J Fox Foundation for Parkinson’s Research to be USD 52 billion in the United States alone. Currently, no effective cure exists, but available PD medical treatments are based on symptomatic prescriptions that include drugs, surgical approaches and rehabilitation treatment. Due to the complex biology of a PD brain, the design of clinical trials and the personalization of treatment strategies require the identification of accessible and measurable biomarkers to monitor the events induced by treatment and disease progression and to predict patients’ responsiveness. In the present review, we strive to briefly summarize current knowledge about PD biomarkers, focusing on the role of extracellular vesicles as active or involuntary carriers of disease-associated proteins, with particular attention to those research works that envision possible clinical applications.

## 1. Parkinson Disease

First described by James Parkinson in 1817, Parkinson’s disease (PD) is today the most common neurodegenerative disease after Alzheimer’s disease [1]. According to the World Health Organization (WHO) definition, PD is “a degenerative condition of the brain associated with motor symptoms (slow movement, tremor, rigidity, walking and imbalance) and a wide variety of non-motor complications (cognitive impairment, mental health disorders, sleep disorders and pain and other sensory disturbances)”. Disability and death due to PD in recent years are increasing faster than they are for any other neurological condition, and the Global Burden of Disease Study 2015 estimates that there may be nearly 13 million people with PD (pwPD) by 2040 [2], suggesting an increasing social and economic burden on modern societies that has to be faced.

### 1.1. PD Etiology and Subtypes

The causes of PD are still largely unknown; if previously the disease was believed to be caused mainly by environmental factors, nowadays research reveals that the pathology is caused by a complex interplay of genetic factors and environmental triggers. Histologically, PD is characterized by the prominent death of dopaminergic neurons, especially in the substantia nigra pars compacta (SNpc) area of the brain, and by the presence of Lewy bodies and Lewy neurites, which are inclusions of abnormal aggregates of α-synuclein (α-syn) in neuronal cell bodies and neuronal cell processes, respectively [3]. The study of familial PD has led to the identification of rare mutations in different genes responsible for the monogenic forms of PD, which account for <10% of total PD cases [4]. These genes are SNCA (α-syn), LRRK2 (leucine-rich repeat kinase 2), PARK2 (parkin), PINK1 (PTEN-induced putative kinase 1), VPS-35 (vacuolar protein sorting-associated protein 35), PARK7 (DJ1) and GBA (glucocerebrosidase) [5]. The remaining 90% of patients are considered to suffer from “idiopathic” PD. These cases are sporadic and for them a monogenic inheritance pattern cannot be identified; these cases of PD are considered the result of the interaction between environmental and genetic factors. The genetic background of idiopathic PD is complex, similar to many other common multifactorial conditions, and is characterized by the presence of variants that include single-nucleotide polymorphisms (SNP) and structural variants (microsatellites, minisatellites, insertions, and deletions) that together determine an individual’s susceptibility to disease. Meta-analyses of several different genome-wide association studies (GWAS) have identified 41 PD risk loci [6,7] that are common genetic variants conferring an increased risk of developing the disease. Among environmental triggers, the following deserve to be mentioned: exposure to the heroin side product 1-methyl-4-phenyl-1,2,3,6-tetrahydropyridine (MPTP) [8] or to pesticides such as rotenone, paraquat, organophosphates, and pyrethroids [9], head trauma [10], and central nervous system (CNS) infections [11]. Notably, moderate nicotine and caffeine consumption, and use the of non-steroid anti-inflammatory drugs have been reported to be protective against PD [12].

### 1.2. PD Diagnosis

The clinical diagnosis of PD, especially in the early phases, is still challenging because of the unavailability of specific diagnostic tests. Diagnosis primarily depends on clinical symptoms, along with detecting SNpc neurodegeneration and observing Lewy pathology. Typically, a confirmation of these findings takes place during a post-mortem pathological examination [13]. Studies report that in only 80–90% of PD cases is the diagnosis confirmed at autopsy [14]. The first step of diagnosis is the clinical establishment that the patient is affected by “parkinsonism”. This relies on the three key elements, bradykinesia, tremor and rigidity, with bradykinesia being the essential element, accompanied by the presence of at least one of the other two [15]. Besides these evaluations, of critical importance are also exclusion criteria including the absence of a response to high-dose levodopa despite moderate disease severity, normal “DAT scan” functional imaging, and cerebellar abnormalities. Supportive criteria are a clear beneficial response to dopaminergic therapy, levodopa-induced dyskinesia, rest tremor of a limb, and the presence of olfactory loss [16]. Non-motor features present in the prodromal phase of the pathology also contribute to the diagnosis; these are depression, anosmia, constipation, and REM sleep behavior disorder [17]. Several disorders mimic idiopathic PD, in particular dementia with Lewy bodies (DLB), multiple-system atrophy (MSA), progressive supranuclear palsy (PSP), and corticobasal degeneration (CBD). Moreover, secondary causes can lead to parkinsonism, like drug-induced parkinsonism, as in the case of antipsychotics, toxins, and normal-pressure hydrocephalus, and vascular parkinsonism.

### 1.3. PD Therapeutic Strategies

Currently, disease-modifying treatments for PD are not available, and the drugs focus on controlling the motor symptoms and not on altering the course of the disease. The selective loss of dopaminergic neurons of the SNpc results in dopamine depletion in the striatum [18], hence the mainstay of PD pharmacological treatment is dopaminergic drugs that replace the action of dopamine by activating dopamine receptors, the provision of a precursor that is metabolized to dopamine (e.g., levodopa), or the prevention of the breakdown of endogenous dopamine (e.g., monoamine oxidase-B (MAO-B) inhibitors and catechol-O-methyl transferase inhibitors (COMT) [19,20]. Treatments are tailored to each patient considering the benefits and the side effects, mostly relying on the personal expertise of the neurologist and a trial and error process.

Some novel and promising approaches are currently under investigation including stem cell-based treatments [21], gene therapy [22] and therapies with the aim of contrasting α-syn toxic effects by inhibiting α-syn production, increasing the degradation of α-syn aggregates, and reducing the uptake of extracellular α-syn by neighboring cells [23]. Targeted rehabilitative programs for motor function, swallowing difficulty, and speech disorders are assuming an important role in improving the quality of life of pwPD [24].

As previously explained, there are still many challenges to be overcome regarding PD from the fine understanding of the biological processes and molecular pathways at the basis of the disease, and the discovery of solid and reliable diagnostic and prognostic biomarkers—to be used especially in the prodromal and early phases of the disease—to the development of disease-modifying treatments. We will now review the attractive role of extracellular vesicles (EVs), the state of the art, the need to define laboratory technique guidelines, and future opportunities and perspectives. 

## 2. Extracellular Vesicles

**Figure 1:**
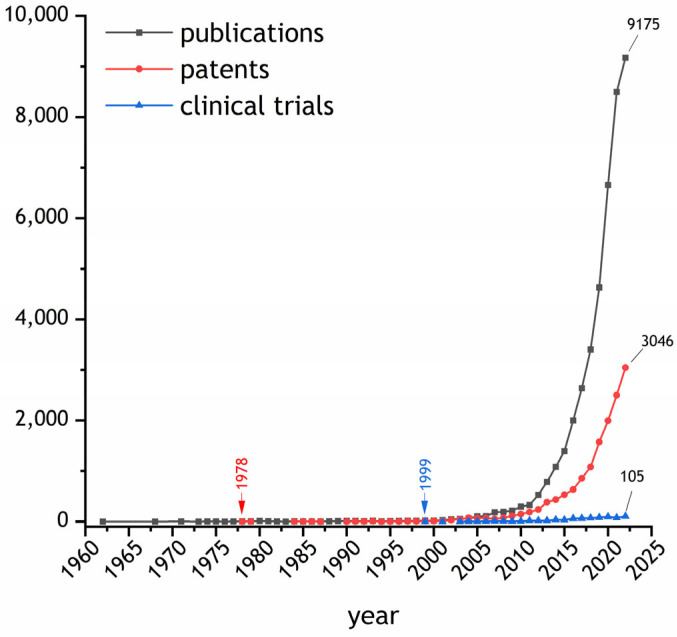
Schematic representation of the exponential growth of the number of publications (grey line) and patents (red line) reported on the Scopus database (https://www.scopus.com/; accessed on 31 December 2022). The blue line indicates the number of registered trials on clinicaltrials.gov. The search for publications patents and trials involved using the following keywords: “extracellular vesicle” OR “exosome” OR “microvesicle”.

EVs is a general term that refers to a complex multitude of “particles that are released from cells, are delimited by a lipid bilayer, and cannot replicate on their own” [25] that can be detected in the interstitial fluid as well as in all biofluids, including blood, saliva, urine, milk, and intraperitoneal fluids [26]. EVs were first described in the 1960s and since their discovery, they have undergone a transformation in the definitions of their structure and role. Initially, they were assumed to be the result of membrane turnover and a waste removal pathway. Later, their release mechanisms were investigated, shedding new light on their function and role with the description of the formation of intraluminal vesicles (ILVs) in late endosomes by the inward budding of the endosomal limiting membrane, followed by the fusion of so-called multivesicular bodies (MVBs). In 1996, Raposo et al. reported the immune-modulating activity of B cell-derived EVs, suggesting new biological implications of these vesicles, far beyond a mere recycling circle [27]. Since then, the investigation of the mechanisms underlying EV release, their classification and nomenclature, as well as their involvement in physiological and pathological cell functions has dramatically increased. The technological evolution that has been accompanying the study of EVs has progressively increased our knowledge about EV heterogeneity in size, structure, and functions in physiological and pathological conditions [28]. This led to an exponential increase in EV-related scientific publications, patents, and clinical trials in the first two decades of this century (Figure 1) as well as to the appearance of EV-focused journals and scientific communities of national and international interest. 

In the current review, our aim is to emphasize the evidence concerning EVs in PD research. For this reason, we will broadly refer to EVs as a wide family, without going deeply into the numerous distinctions related to their biogenesis. However, it is important to acknowledge that while this overarching term encompasses a diverse range of vesicles, it might obscure variations in their origins, physical and chemical properties, and functions. These distinctions, unraveled over time thanks to innovation and technological advancements, could also underlie certain controversies and conflicting evidence that will be later described.

## 3. Extracellular Vesicles in Parkinson’s Disease

### 3.1. Pathophysiology: Spread of Pathology

As mentioned, one of the hallmarks of PD is the aggregation and spread of cytotoxic forms of α-syn. Starting from this premise, several studies have tried to understand if EVs might have a role in either the sequestration and removal or in the transport across the body of misfolded and aggregated α-syn. The reasons for the toxicity of α-syn in PD are complex and involve multiple mechanisms, including the loss of function of aggregated proteins, mitochondrial dysfunction, interference with axonal transport, proteasomal inhibition, synaptic toxicity, and endoplasmic reticulum stress. Mounting evidence suggests that EVs have a role in the propagation of aggregated proteins, in particular in the prion-like propagation of aggregated α-syn, and predominantly in the intercellular spread of aggregates. 

One of the first observations proposing EV-mediated transport as one (but not a unique) form of transport for α-syn was made by Emmanouilidou and colleagues [29], who observed that in vitro-cultured α-syn-expressing cells could release the α-syn monomeric and oligomeric forms both when free in the medium and within exosomes, in a calcium-dependent manner. Subsequently, it was shown that lysosomal dysfunction—a PD relevant stress condition—increases the release of α-syn-containing vesicles that cause toxicity in the recipient cells [30,31]. Further investigations on the mechanisms of the vesicle-mediated release of α-syn in the extracellular space proposed ubiquitination [32] and sumoylation [33] as possible mechanisms involved in the inclusion of α-syn into multivesicular bodies and, thus, in exosomes. It was also found that cells take advantage of the exosomal secretion of α-syn to discard α-syn in specific conditions, i.e., when autophagy is impaired [34]. Nonetheless, criticisms were raised about the impact of EV transport on α-syn release as some in vitro works hypothesized that less than 2% of α-syn is found in association with exosomes [35]. Indeed, even though the percentage of α-syn released by brain and non-brain cells might be small, the specific composition of EVs seems to have a critical role in the pathogenic mechanisms underlying PD. 

Although the mechanisms of EV α-syn loading remain to be elucidated, it was reported that the α-syn–membrane interaction has a role in the conformational changes of α-syn that might alter the protein function and/or drive aggregation in PD development. At the same time, the targeting of α-syn in its different conformations (especially the most neurotoxic ones, i.e., oligomers) at the membrane surface can alter lipid composition because of its ability to disrupt biological membranes [36]. Studies focusing on the chemo-physical interaction of α-syn and membrane components demonstrated that the protein adopts an α-helix conformation when interacting with the cell membrane, in particular with some phospholipids, including phosphatidylserine and phosphatidyl inositol [37]. The α-helix remains entirely buried within the depth of the membrane, whereas the rest of the protein segments present lower membrane penetration and higher flexibility [36], influencing α-syn’s role in vesicle biogenesis and recycling. Data suggest that monomer binding to the membrane is accompanied by the further clustering of α-syn, which can perturb lipid membranes, in particular nanosized vesicles like EVs [38]. The aggregated and toxic form of the protein, oligomeric α-syn, was found to interact with lipids causing structural changes in lipid membranes, leading to membrane disruption, membrane thinning, pore formation, and lipid clustering. Membrane-associated oxidative stress further promotes α-syn aggregation inside the cells, with the consequent release of toxic α-syn species in EVs, both inside and on the surface of the vesicles, propagating the neurodegenerative process to adjacent neurons [39].

EVs derived from pwPD were proven to be enriched in α-syn and to be able to induce the oligomerization of α-syn in a dose-dependent manner [40]. Exogenous α-syn strains can also seed the assembly of endogenous α-syn and its propagation, leading to long-term functional effects [40]. Furthermore, α-syn strains display differential seeding capacities and elongation processes and induce a strain-specific pathology and specific neurotoxic phenotypes, possibly explaining the heterogeneity in the spread and in the symptoms of the pathology [40].

What is of particular interest in the investigation of the role of EVs in PD progression is the observation that α-syn assemblies cross the blood–brain barrier (BBB); intravenous α-syn injection results in its dissemination in the CNS [38]. This can be mediated by EV transport, which is facilitated by the leakage of the BBB, as seen in pwPD [41]. Microglia are also known to play a major role in the neuroinflammation that accompanies the neurodegenerative process. In pwPD, microglia were shown to fail to clear and/or to promote the release of EVs that contained toxic forms of α-syn or other pathogenic factors, possibly potentiating neurodegeneration [42].

Although α-syn is the most studied player in the pathogenic mechanisms of PD, other genes are mutated in familial PD, and other proteins were found to be associated with EVs in pwPD. For example, mutant LRRK2 acts at multiple points within the endosomal pathway and thus has the potential to modulate exosome biogenesis. It was shown that LRRK2 is released within EVs and regulates their biogenesis [43], with LRRK2-loaded EVs being also found in the urine of pwPD [44]. Similarly, the microtubule-stabilizing protein Tau is known to become hyperphosphorylated and aggregation-prone in so-called taupathies, including PD. Different mechanisms, including EV transport, allow brain tau to cross the BBB and circulate in the bloodstream, where it can associate to exosomes, and in particular with neuron-derived exosomes expressing the L1CAM protein marker [45].

Finally, the oxidative stress of neurons is crucial in PD progression, contributing to the cellular dysfunction of neurons. In this regard, studies performed on preclinical models of PD have demonstrated that EV-associated microRNAs have an impact on brain tissue. In particular, the inhibition of exosomal miR-137 relieved oxidative stress, promoted neuron viability, and inhibited the apoptosis of neurons by up-regulating oxidation resistance 1 (OXR1), which inhibits oxidative DNA damage [46].

### 3.2. Biomarkers for Diagnosis and Prognosis

Over the years, accumulating evidence has demonstrated that EVs are crucial for intercellular communication within the brain, and it is commonly accepted that EVs can move from the bloodstream to the CNS crossing the BBB, even though the mechanisms of this transfer are largely unclear. The possibility to monitor and observe the processes occurring within the brain taking advantage of complex multifaceted nanovesicles, i.e., EVs, has inspired a huge body of literature about brain disorders including neurodegenerative diseases where the disruption of the BBB facilitates the transfer of EVs from the systemic circulation to the brain and vice versa [47]. EVs and their constituents, either proteins, nucleic acids, lipids or small metabolites, have been the object of several studies that over the years have tried to identify molecular biomarkers for the diagnosis of PD.

One of the first studies performed on the blood of pwPD to verify the actual potential of EVs as biomarkers of PD was attempted by Shi and colleagues, who demonstrated that both plasma exosomal α-syn [48] and tau [45] correlated with PD severity, performing even better than the quantification of free α-syn or tau in cerebrospinal fluid (CSF). First, a large cohort of 267 PD and 215 age- and sex-matched healthy controls were considered and α-syn concentration was assessed in plasma L1CAM-containing (L1CAM+) exosomes (using Luminex assays). No significant difference in the total plasma α-syn concentrations was observed in PD versus controls, but the α-syn concentration in plasma L1CAM+ exosomes was significantly higher in pwPD compared to that in healthy controls, with the plasma exosomal α-syn/total α-syn ratio being also significantly higher in pwPD than in controls [48]. The ROC curve revealed moderate diagnostic performance for α-syn within L1CAM+ EVs (area under the curve/AUC = 0.654) as well as for the exosomal α-syn/total α-syn ratio (AUC = 0.657). Interestingly, significant correlations were found between plasma α-syn in L1CAM+ exosomes and the plasma exosomal α-syn/total α-syn ratio with UPDRS motor scores (UPDRS III) [48]. These data were confirmed by the observation that neuronal (L1CAM+) EVs can predict and differentiate pwPD from atypical parkinsonisms. Using a combined protocol of immunoprecipitation and electrochemiluminescence assays, it was established that an α-synuclein level in L1CAM-immunocaptured exosomes of above 14 pg/mL is a robust biomarker for the differential diagnosis of PD and atypical parkinsonisms (ROC AUC = 0.98) [49]. Similarly, it was reported by Cerri and coauthors [50] that the quantification of plasma exosomal α-syn (not only L1CAM+) normalized for total α-syn (whole plasma) via ELISA was significantly increased in pwPD (39 pwPD and 33 healthy controls) compared to that in controls. The results were also correlated with the measures of disease severity, indexed by the UPDRS III and Hoehn and Yahr (H&Y) scores, as well as with the Glucocerebrosidase (GCase) activity measured in lymphocytes [50]. This study is in line with other data that demonstrated the abundance of α-syn not only in neuronal circulating EVs, but also in non-brain EVs [51]. In particular, it was reported that the quantification of plasma EV-associated α-syn (without prior enrichment for brain-derived EVs) was effective in distinguishing pwPD (n = 94) from people with DLB (n = 48) and PSP (n = 49). ROC analysis showed an AUC of 0.804 for PD and DLB and an AUC of 0.815 between PD and PSP, whereas the ROC AUC was 0.769, comparing pwPD and control subjects. A significant inverse correlation was also observed with UPDRS III and H&Y scores [51].

Interestingly, EVs from non-brain cells were found to be loaded with α-syn in pwPD, as were brain-derived EVs, for example those from red blood cells [52] and peripheral lymphocytes [53]. This observation can partially explain the better performance of the quantification of α-syn in the entire population of blood-derived EVs compared to that of its quantification in specific neuronal populations. Moreover, L1CAM is known to identify vesicles released by neurons, although its specificity is controversial and not all of the neuronal EVs will be loaded with L1CAM. It was also shown that the blood concentration of EVs derived from neurons (SNAP-25), astrocytes (EAAT1), and oligodendrocytes (OMG) is significantly augmented in pwPD compared to those in other forms of parkinsonisms, with good diagnostic potential reported for these EV subfamilies (AUCs of the ROC curve for plasma neuron-, astrocyte- and oligodendrocyte-derived EVs were 0.82, 0.75, and 0.78, respectively) [54]. 

A comprehensive analysis of neuron-derived EVs (L1CAM+) in pwPD (n = 32) showed that the toxic oligomeric form of α-syn is significantly increased in L1CAM+ EVs, whereas total α-syn, STX-1A, and VAMP-2, proteins that are known to be involved in synaptic function, are decreased [55]. The analysis of the diagnostic performance of the proposed biomarkers in L1CAM+ EVs showed good performance for oligomeric α-syn (ROC curve AUC = 0.824); the results were even better when two biomarkers were considered in L1CAM+ EVs: the oligomeric α-syn/STX-1A ratio (AUC = 0.871) and oligomeric α-Syn/VAMP-2 ratio (AUC 0.876). Importantly, also in this study, the amount of the oligomeric form of α-syn in L1CAM+ EVs correlated with disease duration and clinical severity (UPDRS III and H&Y Scales) [55]. The same protocol was also applied for the combined quantification of oligomeric α-syn and aggregated tau in L1CAM+ EVs comparing pwPD (n = 70) and atypical parkinsonisms. Even though in this case the diagnostic performance did not include control subjects, the diagnostic performance of the combination between the two biomarkers (oligomeric α-syn/Tau aggregates ratio) showed far better diagnostic performance than did that of the single biomarker, with ROC analysis obtaining AUC = 0.902 for pwPD compared to CBD and AUC = 0.908 for pwPD compared to PSP [56].

Following a similar approach of exosomal quantitation [48] but using a different technology, single-molecule array (SiMoA) assays, Shi and colleagues evaluated the possibility of using tau as a PD biomarker, measuring its concentration both in whole plasma and L1CAM+ plasma exosomes in pwPD, Alzheimer’s disease (AD), and healthy controls [45]. Plasma exosomal tau was significantly higher in PD than in healthy controls, but was not significantly different in AD patients; importantly, exosomal tau correlated with CSF tau concentration [45]. The diagnostic performance, as evaluated via ROC analysis, showed that L1CAM+ exosome-associated tau was modestly predictive in distinguishing between PD and healthy controls (AUC = 0.607), and was associated with disease duration but not with other clinical scales in pwPD [45].

Taken together, these data suggest variations in the subpopulations of vesicles circulating in the blood of pwPD that might relate not only to a single protein variation, but possibly to multiple factors associated with EVs. In line with this observation, Tomlinson and colleagues performed a proteomic analysis of serum EVs and found that, despite the absence of significant differences in EV numbers or sizes, 23 EV-associated proteins were differentially abundant in PD [57]. Subpopulations of vesicles that are either differentially regulated or enriched for certain proteins (including but not limited to α-syn) in response to the disease process could explain this observation. An overall biochemical characterization of EVs was proposed as a potential biomarker for PD in 2019 [58]. Taking advantage of the Raman spectroscopy approach, serum-derived EVs from a small cohort of pwPD (n = 22) and healthy controls (n = 18) were analyzed; the results identified a specific biochemical fingerprint for pwPD [58]. Although the complexity of the Raman spectrum did not allow the complete definition of the molecules responsible for the observed differences, the ROC curve demonstrated moderate diagnostic accuracy (AUC = 0.71) and a correlation between the biochemical features of serum EVs and the clinical scales used to profile pwPD (UPDRS III and H&Y scores) [58].

Of course, EVs are carriers of multiple bioactive compounds—other than proteins—that can play a role as PD biomarkers, and among them miRNAs have received increasing attention. The first observation in this topic focused on EV-associated miRNAs isolated from CSF. Gui and colleagues demonstrated that five miRNAs that circulate in CSF in association with EVs could discriminate PD from healthy controls with an accuracy calculated by the ROC curve of higher than 90%, with the highest performance being achieved with miR-409-3p. Notably, the combination of miR-153 and miR-409-3p in CSF-derived EVs could enhance the performance of discrimination significantly (AUC = 0.99) [59]. In blood, miR-331-5p and miR-505 were found to have optimal diagnostic performance (AUC > 0.85) between pwPD and healthy subjects [60]. In a different cohort, the downregulation of miR-19b and upregulation of miR-195 and miR-24 were found to have consistent diagnostic potential with variable accuracy ranging from 70 to 90% compared to that in healthy subjects (n = 40) [61].

Recently, the search for PD biomarkers has expanded towards organic fluids other than blood and CSF. Urine and saliva are intriguing sources of biomarkers for chronic pathologies like PD, and measurements performed in accessible biofluid would allow clinicians to periodically monitor disease progression and therapy effectiveness, without the need for blood and CSF collection. Studies performed on urinary samples of pwPD showed that EVs loaded with elevated levels of autophosphorylated Ser(P)-1292 LRRK2 could be detected in people with idiopathic PD [44,62]. SNAP23 and calbindin were found to be elevated as well in urinary EVs from pwPD compared to healthy subjects, with both markers having moderate diagnostic accuracy after ROC curve calculation (AUC = 0.68 for SNAP23 and AUC = 0.75 for calbindin; AUC = 0.76 for the combination of calbindin and SNAP23 in the same logistic model) [63] (Table 1).

Saliva has received attention in the field of neurodegenerative disease diagnostics as it shares multiple components with plasma, is collected in non-invasive ways, and has the advantage of a reduced interference of lipoproteins. A pilot study performed on pwPD showed an increased level of L1CAM and phosphorylated α-syn in salivary EVs compared to those in healthy controls, suggesting salivary EVs as a source of PD biomarkers [64].

### 3.3. Therapy

As mentioned above, current therapies for PD are far from optimal, treating the symptoms of the disease but being unable to alter the undergoing neuronal damage. One of the main problems with OD drugs is that they must cross the BBB, a natural dynamic barrier composed of specialized epithelial cells that together with pericytes and astrocytes form a tight barrier that regulates the flow of substances into the brain, including therapeutic agents. Different strategies have been designed in an attempt to bypass this obstacle [65], including the use of nanocarriers that may be of synthetic or biological origin.

EVs, due to their properties, are emerging as a new class of nanocarriers of drugs and therapeutic molecules (proteins—including growth factors—lipids, various DNA and RNA species, etc.) that could be used in neurodegenerative diseases including PD [66]. EVs-based drug design could be divided into two main groups: the first takes advantage of the paracrine effect of EVs as part of the “secretome” of cell therapies (e.g., mesenchymal stem cell (MSC), and the second involves the use of EVs as drug delivery systems to deliver biomolecules and drugs to recipient cells.

In the first case, the intrinsic therapeutic effect of stem cell-derived EVs is exploited: the therapeutic effect of MSCs is mainly mediated by soluble factors, among which EVs play a crucial role [67]. Moreover, the use of MSC-derived EVs is safer than that of MSCs themselves, which presents the risk of malignant transformation [68]. Several promising in vitro and in vivo experiments were carried out: neural stem cell (NSC) EVs attenuated the ROS-induced apoptotic pathway and neuroinflammation in cultured dopaminergic neuroblastoma neurons [69]. The incubation of dopaminergic neuroblastoma neurons exposed to 6-OHDA neurotoxin and umbilical cord MSC-EVs increased cell viability and proliferation, and inhibited apoptosis by inducing autophagy. Furthermore, in vivo, these EVs reduced the loss of dopaminergic neurons in the *substantia nigra* and upregulated dopamine in the striatum in a 6-OHDA-induced PD rat model [70]. EVs derived from human exfoliated deciduous teeth (SHED) were also shown to suppress 6-OHDA-induced dopaminergic neurons apoptosis [71]; the same EVs administrated intranasally in a PD rat model increased tyrosine hydroxylase expression in the *substantia nigra* and improved gait parameters [72].

Biological EVs also represent a potential avenue for drug delivery systems; notably, they can also overcome the drawbacks of synthetic nanovectors: allergic reactions and off-target accumulation in the liver and spleen. In addition, biological EVs can bypass the macrophagic system due to the presence of CD47 surface molecule [73,74], are highly biocompatible and stable, and are less immunogenic than are synthetic nanovectors. Therapeutic molecules can be loaded in EVs, both modifying the parent cells before isolation and altering EVs after isolation. In addition, EVs’ protein surface composition can be modified to enhance their ability to cross the BBB [48]. Rabies virus glycoprotein (RVG)-modified EVs loaded with α-syn siRNA were administered into the brain of PD mice; this reduced total and aggregated α-syn in *sustantia nigra* dopaminergic neurons [75]. Izco et al. improved the system by increasing the efficacy of siRNA treatment using short hairpin RNA microcircles, and obtained a decrease in α-syn aggregation, a reduced loss of dopaminergic neurons, and an improvement in clinical symptoms in an α-syn PFF intrastriatally injected PD mouse model [76]. Several small molecules have also been encapsulated into EVs; EVs loaded with curcumin, an anti-inflammatory compound, were administered intranasally in different neurological disease models. The results showed an inhibition of inflammation and an increased apoptosis of IL1β^+^ microglia [77]. Finally, dopamine, which cannot cross the BBB when administered intravenously [78], improved functional recovery in a murine PD mouse model without any toxicity when loaded in blood EVs [79].

EV-based therapies, in general, have drawn a lot of interest; however, this field is still in its infancy and many problems have yet to be solved, including the large-scale production of highly purified EVs, standardized methods of purification and characterization, the efficiency of drug encapsulation, and knowledge of their mechanisms of action [80].

## 4. Clinical Translation and Future Perspective

From the above-mentioned results, it is apparent that EVs are valuable candidates for the identification of biomarkers to allow the early diagnosis of PD and to differentiate between different tauopathies, as well as having possible usefulness as therapeutics. However, it must be noted that the huge body of evidence that emerged from the literature of the last decades has not led to a clinical translation of the proposed biomarkers, with only a few clinical trials registered (Table 2).

The reasons for the discrepancy between research findings and clinical trials can possibly be found in the lack of standard procedures related to the laboratory activities of EV isolation, characterization, and storage that limit the reproducibility and clinical translation of the procedures. Indeed, several variables are known to impact the downstream results of EV analysis, including, for example, storage conditions that can affect EVs concentration, physical properties, and functionality [81]. The EV scientific community, represented by the International Society for Extracellular Vesicles (ISEV), is active in monitoring the current literature, creating dedicated task forces, and releasing guidelines and recommendations [25,82,83,84,85]. No consensus, though, has been reached on standard operating procedures in the isolation and characterization of EVs from liquid biopsies, because of a number of problems including the heterogeneity of EV themselves, the variable EV sources, and the plethora of techniques used to detect EV related molecules like protein markers and nucleic acids [86]. Moreover, the increasing knowledge of EV biogenesis and function led to changes in the design of EV analyses, which can now focus on studying single vesicles and pure preparations, or can even concentrate on the biomolecular corona that can be co-isolated with EVs. Technical limitations and a small sample size, together with a lack of replicates and standard operating procedures, are often the basis of the lack of translation of the research results to clinics.

From a methodological point of view, difficulties in the quantification of biomarkers like α-syn in EVs can be encountered in several steps of the analytical procedure. As an example, some concerns were raised on the specificity of one of the most widely used neuronal biomarkers, L1CAM [87], although several studies could successfully enrich brain EVs using this protein, confirming its specificity [88]. In the future it will be essential to identify and standardize robust biomarkers and methods to identify the exact cellular origin of EVs. Similarly, criticisms were made for some commercial kits for the preparation of EV suspensions that might lead to the co-isolation of non-specific molecules [89]. Indeed, the standardization of isolation and characterization methods will be crucial to improving the reproducibility of the results and their actual translation to clinics.

Clinically, little information is available on EVs, including their half-life in biological fluids and whether or not biological fluids can contain levels of disease-associated EVs that are suitable for the detection range of current technologies [90]. 

Among the obstacles to the validation of specific PD biomarkers, the selection of the analyzed cohort of subjects and improvements in clinical study design could significantly improve the translational ability of the studies in this field. As highlighted in Table 1, most works cited in the present review were performed in very small groups of subjects, which is reasonable for pilot studies demonstrating a proof of concept, but were not further validated on larger cohorts. Studies performed in larger groups of patients in different disease stages will nevertheless be needed to validate the findings and to better evaluate potential correlations with motor impairment and disease progression [51], avoiding misleading conclusions. Additionally, authors developing new techniques and assays should be alerted about the need for different control groups in their studies when designing a trial. Indeed, pilot studies are usually performed on disease cohorts, PD in this case, which are compared to healthy sex- and age-matched groups, but ROC results can be an overestimate of the real potential of the diagnostic assay due to the lack of an experimental group that shares the comorbidities of pwPD.

Finally, it has to be noted that the tremendous potential of new techniques and methods should be always cautiously assessed in single-center studies. The lack of EV standardization for isolation and characterization methods limits the reproducibility of results, as was recently reported [84]. For this reason, data should be validated with multicenter studies or studies that take advantage of biobanking procedures as they rely on standard operating procedures, whereas single-center studies mainly rely on a few operators and one single laboratory.

## 5. Conclusions

Taken together, the data summarized in the present review lead us to two main conclusions: (i) EVs have remarkable potential in PD diagnosis, prognosis and treatment, specifically because of their multifaceted nature and their ability to co-transport multiple biomarkers providing a snapshot of PD progression; (ii) the path towards clinical validation is still tortuous as some pieces of the puzzle (representing a knowledge gap) as well as standardized highly sensitive techniques (representing a technological gap) are still missing. Although both knowledge and technological gaps might require quite a lot of time and efforts to be fulfilled, in our opinion, three main solutions could significantly impact the field in the near future: the accurate selection of a cohort; the design of multicenter studies; the standardization of methods. If the latter cannot be achieved, detailed reporting could be significantly beneficial for translation to clinics.

## Figures and Tables

**Table 1:** Summary of the study on PD biomarkers with reported data on clinical samples. Only studies with reported clinical power calculations are included. Abbreviations: AD: Alzheimer’s disease; AUC: area under the curve after ROC curve calculation; CBD: corticobasal degeneration; CSF: cerebrospinal fluid; DLB: dementia with Lewy bodies; ECL: electrochemi-luminescence; HC: healthy control; MSA: multiple-system atrophy; PD: Parkinson’s disease; PSP: progressive supranuclear palsy; SEC: size exclusion chromatography.

*Biomarker*	Source	EV Subtype	Isolation Method	Considered Population	Detection Method	Diagnostic Performance	Ref.
Blood							
α-syn	serum	L1CAM+	Immunoaffinity	PD = 290 MSA = 50 PSP = 116 CBD = 88 HC = 191	ECL	AUC = 0.86 (PD vs. HC); AUC = 0.98 (PD vs. MSA); AUC = 0.94 (PD vs. PSP + CBD)	[49]
	plasma	L1CAM+	Immunoaffinity	PD = 267 HC = 215	Luminex^®^ (fluorimetric)	AUC = 0.654	[48]
	plasma	Total EVs	SEC	PD = 96 DLB = 50 PSP = 50 HC = 42	ECL	AUC = 0.804 (PD vs. DLB); AUC = 0.815 (PD vs. PSP); AUC = 0.769 (PD vs. HC)	[51]
Oligomeric α-syn; STX-1A; VAMP2	serum	L1CAM+	Immunoaffinity	PD = 32 HC = 40	ELISA	oligomeric α-syn: AUC = 0.820; oligomeric α-syn/STX1: AUC = 0.871; oligomeric α-syn/VAMP2: AUC = 0.876	[55]
Oligomeric α-syn; Tau aggregates	serum	L1CAM+	Immunoaffinity	PD = 70 PSP = 21 CBD = 19	ELISA	oligomeric α-syn/Tau aggregates: AUC = 0.902 (PD vs. CBD) Tau aggregates: AUC = 0.908 (PSP vs. PD)	[56]
Tau	plasma	L1CAM+	Immunoaffinity	PD = 91 AD = 106 HC = 106	SiMoA	AUC = 0.607 (PD vs. HC)	[45]
EV count	plasma	SNAP25+ EAAT1+ OMG+	Immunoaffinity	PD = 15 MSA = 15 PSP = 7 HC = 15	ELISA	SNAP25+ EVs: AUC = 0.82 (PD vs. HC); EAAT1+ EVs: AUC = 0.75 (PD vs. HC) OMG+ EVs: AUC = 0.78 (PD vs. HC)	[54]
Raman spectrum	serum	Total EVs	SEC and ultracentrifugation	PD = 22 HC = 19	Raman spectroscopy	AUC = 0.71	[58]
miR-331-5p miR-505	plasma	Total EVs	Polymer based precipitation and Targeted filtration	PD = 52 HC = 48	RT-qPCR	miR-331-5p: AUC = 0.849; miR-505: AUC = 0.898	[60]
miR-19b miR-24 miR-195	serum	Total EVs	Polymer based precipitation	PD = 109 HC = 40	RT-qPCR	miR-19b: AUC = 0.753; miR-24: AUC = 0.908; miR-195: AUC = 0.697	[61]
Other biofluids							
miR-409-3p miR-153	CSF	Total EVs	Ultracentrifugation	PD = 47 HC = 27	RT-qPCR	miR-409-3p: AUC = 0.90; miR-153 and miR-409-3p: AUC = 0.99	[59]
SNAP23 calbindin	urine	Total EVs	Ultracentrifugation	Discovery cohort PD = 28 HC = 22	Mass spectrometry	Discovery Cohort SNAP23: AUC = 0.80 Calbindin: AUC = 0.75	[63]
				Replication cohort PD = 57 HC = 51		Replication cohort SNAP23: AUC = 0.68 Calbindin: AUC = 0.75	

**Table 2:** List of the current clinical trials registered on clinicaltrial.gov on the use of EVs as a biomarker or as a treatment for PD.

NCT Number	Study Title/Funding	Study Type	Intervention	Single (S) or Multicenter (M)	Locations	Link
NCT05320250	Saliva and Extracellular Vesicles for Parkinson’s Disease (RaSPiD)	Observational	PROCEDURE: Saliva collection	M	Italy	https://clinicaltrials.gov/study/NCT05320250 *
NCT05871359	Transcranial Direct Current Stimulation and Dual Tasks (Tdcs&DT)	Interventional	DEVICE: Transcranial Direct Current Stimulation PROCEDURE: Dual task	S	Italy	https://clinicaltrials.gov/study/NCT05871359 *
NCT05452655	Intensive Multidisciplinary Rehabilitation and Biomarkers in Parkinson’s Disease	Interventional	BEHAVIORAL: Multidisciplinary Intensive Rehabilitation vs. Muscle-stretching and active mobilization exercises	S	Italy	https://clinicaltrials.gov/study/NCT05452655 *
NCT03775447	Fox BioNet Project: ECV-003	Observational	PROCEDURE: Lumbar Puncture	M	Canada, United States of America	https://clinicaltrials.gov/study/NCT03775447 *
NCT05902065	Effect of a Progressive Treadmill Training Protocol for Parkinson’s Disease	Interventional	DEVICE: AVR Treadmill training with C-Mill vs. Conventional Treadmill training with C-Mill	S	Italy	https://clinicaltrials.gov/study/NCT05902065 *
NCT04603326	FoxBioNet: ECV (Extracellular Vesicle) 004	Observational	PROCEDURE: Lumbar Puncture	M	Canada, United States of America	https://clinicaltrials.gov/study/NCT04603326 *
NCT05807581	Clinical, Molecular and Electrophysiological Profiling of Parkinson’s Disease: the Role of Non-pharmacological Therapies	Interventional	OTHER: physical activity vs. iTBS	M	Italy	https://clinicaltrials.gov/study/NCT05807581 *
NCT04350177	A Study to Assess Single and Multiple Doses of IkT-148009 in Healthy Elderly Participants and Parkinson’s Patients	Interventional	DRUG: IkT-148009 vs. Placebo	M	United States of America	https://clinicaltrials.gov/study/NCT04350177 *
NCT01860118	LRRK2 and Other Novel Exosome Proteins in Parkinson’s Disease	Observational		S	United States of America	https://clinicaltrials.gov/study/NCT01860118 *
NCT03415984	Prevalence of Age Related Macular Degeneration (ARMD) in Parkinson’s Patients and Assesment of the Role of L-DOPA (AMD-PARK)	Observational	DIAGNOSTIC_TEST: Color retinograph; Optical coherence tomograph; Fundus autofluorescence imaging	S	France	https://clinicaltrials.gov/study/NCT03415984 *
NCT05815524	Physical Activity in Patients With Parkinson’s Disease: a “Disease Modifying” Intervention?	Interventional	OTHER: Physical activity training	S	Italy	https://clinicaltrials.gov/study/NCT05815524 *
NCT05109364	Terazosin and Parkinson’s Disease Extension Study	Interventional	DRUG: Terazosin	S	United States of America	https://clinicaltrials.gov/study/NCT05109364 *

* accessed on 30 October 2023.

## Data Availability

Not applicable.
